# Physiological outcomes of calming behaviors support the resilience hypothesis in horses

**DOI:** 10.1038/s41598-018-35561-7

**Published:** 2018-11-30

**Authors:** Chiara Scopa, Elisabetta Palagi, Claudio Sighieri, Paolo Baragli

**Affiliations:** 1 Italian National Reference Centre for Animal Assisted Interventions, Istituto Zooprofilattico Sperimentale delle Venezie, Legnaro (Padua), Italy; 2 Unit of Ethology, Department of Biology, University of Pisa, Pisa, Italy; 3 Department of Veterinary Sciences, University of Pisa, Pisa, Italy

## Abstract

To manage a stressful stimulus animals react both behaviorally and physiologically to restore the homeostasis. In stable horses, a stressful stimulus can be represented by social separation, riding discomfort or the presence of novel objects in their environment. Although Heart Rate Variability is a common indicator of stress levels in horses, the behavioral mechanisms concurrently occurring under stressful conditions are still unknown. The sudden inflation of a balloon was administered to 33 horses. Video-recording of self-directed behaviors (snore, vacuum chewing, snort, head/body shaking) and monitoring of heart activity (HR and SDRR) were conducted for five minutes before (Pre-test) and after the stimulus administration (Stress-test). During the Stress-test, only snore and vacuum chewing increased and a significant increase was also recorded in both HR and SDRR. Moreover, the snore variation between the two conditions showed a significant correlation with the variation of both HR and SDRR. With the snore acting as stress-releasing behavior to restore basal condition, the homeostasis recovered via the enactment of such behavior could be physiologically expressed by an increasing vagal activity. Hence, the capacity to maintain homeostasis (resilience) could correspond to a prevalence of parasympathetic control on heart activity, intervening when certain behaviors are performed.

## Introduction

To face a potentially stressful situation an animal can engage in different behaviors. Whatever the reaction, the behavior guiding the animal far from the negative stimulus is strictly linked to specific physiological reactions which are driven by a change in autonomic and neuroendocrine activities^1^. Stress was originally defined as a general non-specific response of the body to any noxious stimulus^2^. Later, Koolhaas and colleagues^3^ underlined that the stress condition could be restricted to those circumstances where an environmental demand exceeds the natural regulatory capacity of an organism, in particular situations that include unpredictability and uncontrollability. Besides, the definition of stress was extended by differentiating between the stressor (i.e. the stimulus that threatens the homeostasis) and the stress response which corresponds to the body reaction that restores the homeostasis^4^. Hence, stress response may be defined in general as nonspecific modification of body functioning not depending on the situation itself, but rather on the negative interpretation that the animal makes of the situation^5^. For example, thanks to different previous experiences and individual plasticity, different subjects can respond in different ways to the same stimulus^6^.

During a stress response an activation of the Sympathetic Nervous System (SNS) occurs, thus promoting fast responding mechanisms to handle a wide range of functions (e.g., respiratory, endocrine, and cardiovascular response). During the activation of these processes, Heart Rate (HR) increases. On the other side of the scale, Parasympathetic Nervous System (PNS) activity slows the heart activity (HR) down, generally regulating bodily function while the animal is at rest^7^. Many different specific stressors may induce a switch of this high sensitive autonomic set of scales towards a prevalent sympathetic or parasympathetic control^8–10^. As a matter of fact, the best way to monitor and measure the balance between these nervous strategies is Heart Rate Variability (HRV), representing the quantitative marker of autonomic system^11^. HRV reflects the fine-tuning of cardiac activity to cope with situational demands^12^ and it has been associated to the emotional regulation ability (i.e. the capacity to process emotional stimuli)^13,14^. In this perspective, HRV has been used for emotional states recognition, reflecting all conditions characterized by high-arousal state, even though it fails to distinguish the valence (positive or negative) of the situation itself^15^. A reduction of some parameters accounting for HRV (such as SDRR or RMSSD) has been often associated to stressful situations or poor health conditions in both human and non-human animals^16–19^. Such reduction indicates a correspondent reduction in dynamic complexity of HRV itself and a reduction in parasympathetic control of cardiac activity. However, all those decreasing parameters refers to more regular HRV series, which are markers of higher sympathetic activity^11,20,21^.

In horses the measure of HRV has been employed to monitor stress levels in several studies^22,23^. The physiological and behavioral stress response is an adaptive mechanism specifically shaped to cope with noxious situations, such as predator attacks, storms, disease, starvation, transportation, and others^24–27^. For example, stable horses may suffer some physical and psychological stress induced by specific activities or by fear and anxiety for novel stimuli, social separation, transportation, pain and discomfort^28^. Due to the broad variety of sports, therapies and recreational activities involving horses, many studies tried to provide a sort of guidelines to make caretakers aware of frustration or stress condition in this species. Moreover, the intimate relationship developed between horses and humans during centuries fostered a flourishing number of studies on the topic.

As it has been already pointed out, a reductionist approach of relying on the measurement of a single biological response as an indicator of stress can be misleading^29^ and, at present, it is known that, in a stressful context, hormonal and behavioral strategies are strictly related to how the horse perceives the stimulus^30^. The behavioral manifestation of frustration should be explored concomitantly with the variation of the physiological parameters in order to reach a holistic interpretation of the internal state of the animal. In horses data are not exhaustive yet; nevertheless some behaviors have been correlated with frustration or motivational conflict in several studies. The *snort*, defined as a loud exhalation through the nostrils, seems to express horses’ restlessness^31^ and frustration^32^. On the other hand, snort has been lately suggested to be a reliable indicator of positive emotions since its production is associated with positive contexts (in pasture, while feeding) and it is less frequent in horses showing an altered welfare^33^. In horses and in other mammalian species the *vacuum chewing* (i.e. chewing without anything in the mouth^34^) is considered a displacement activity performed in stressful situations^32,35–37^. However, this behavior has been also associated to emotions, which have a positive valence, regardless of the arousal level of individuals^38^. Among non-vocal sounds produced via the passage of the air through the nostrils, there is also the *snore* which is defined as a very short raspy inhalation sound produced in a low alert context, such investigating a novel object or obstacle^33^. Finally, *head/body shaking* is considered a stress-related behavior when the rhythmic motion of the head or body occurs repetitively^39^.

Here, in a familiar environment, we administrated a sudden, unfamiliar and unpredictable stimulus to horses and measured the distribution of each of the selected behaviors over time (minute by minute) in order to define a time-window in which the behaviors were statistically more frequent compared to a control period. If the behaviors that significantly varied after the administration of the stimulus (criterion 1) also match with the variation of the sympathetic/parasympathetic control over cardiac activity (criterion 2), those specific patterns could be more reliable than others as indicator of stress in horses. To verify the second criterion, we checked for a possible correlation between the entity of the variation of each tested behavior (e.g., *behavior*_experimental_ minus *behavior*_control_) and the shifting of physiological parameters over cardiac control.

## Methods

### Tested Animals

The experimental design was based on Baragli *et al*.^40^ study, in which any further information about tested animals, experimental design and parameters collected in the present work can be found. From the original sample, we collected data from 33 horses, aged 6–24 from four different stables (for a complete list of the animals considered see Supplementary Table S1). We included in the analysis those animals whose tests presented the same time duration in both control and experimental phases and whose video were recorded. Since it was recently suggested that not only breed but also individual stabling conditions may influence temperament and emotions in horses^41^, animals tested in our study had to fit specific stabling criteria^40^.

### Experimental Design

In accordance with Désiré *et al*.^42^, the general characteristics of the stress response test were defined *a priori* as the sudden appearance of an unfamiliar, unpredictable and intrinsically unpleasant stimulus designed to induce an avoidance reaction. Using a remote-control device, a balloon was suddenly inflated (visual and auditory stimulus) in the horse’s customary environment (its own stall), without the direct intervention of the experimenter. Indeed, when animals respond to situations/stimuli, they experience specific emotional states. Emotions may be defined by two fundamental dimensions: the valence (emotional experiences perceived as negative or positive, rewarding or punishing) and the level of arousal^15,43^. Therefore, emotional responses are activated following potentially rewarding or punishing stimuli, which determine the emotional valence^44,45^. Considering this, we may assume that the appearance of an inflated balloon in the familiar environment could elicit a high-arousal response in horses and it represents a negative situation. To record data on HRV, a Polar RS800 model heart rate monitor (Polar, Kempele, Finland) was fastened to the horse by an elastic chest belt. The heart rate monitor and webcam were synchronized with a chronometer, after which the horse was left alone for 5 mins to become accustomed to the presence of the apparatus in the stall (Pre-Test). The operator then opened the compressed air valve, inflating the balloon, which opened the flaps on the device. The balloon suddenly appeared in the horse’s stall, remaining inflated for 5 mins (Stress Test). Video and heart rate variability recording began at the start of the Pre-Test and lasted for the entire Stress Test^40^.

### Parameters collected and Data Analysis

We analyzed Pre-Test and Stress-Test videos and collected the frequency (number of times in which the behavior was displayed during the test) of all relevant behaviors performed. By paying particular attention to those patterns considered as indicators of frustration we focused on *Snorts (SNT)*, *Snore (SN)*, *Vacuum Chewing (VC)*, *Head/Body Shaking (HBSH)* and *Avoidance/Flee attempts* (for detailed definition see Table 1).

**Table 1:** Description of the behavioral patterns monitored and collected during both the Pre-Test and the Stress-Test.

*Snort* (SNT)	Operational definition	A snort is a forceful exhalation through the nostrils and characterized by an audible flutter pulsation^31^. It has been mostly associated with a hygienic function of clearing the nostrils of phlegm, flies or other irritants^47^.
	Functions suggested	It is used defensively and aggressively and is in equestrian contexts associated with exercise and conflict during restraint^66^. Snorts appear to be a displacement activity and seem to express the horses’ restlessness^31^. and frustration^32^. On the other hand, snort appears as a possible reliable indicator of positive emotions since its production is associated with positive contexts (in pasture, while feeding) and states and it is less frequent in horses showing an altered welfare^33^.
*Snore* (SN)	Operational definition	Snores are non-voiced sounds that seem incidental to inhalation, especially under specific circumstances^47^.
	Functions suggested	The snore that is a broadband inhalation sound can be heard when the horse inhales to emit an alarm blow or has dyspnea lasting 0.3–0.5 seconds^31^. It probably serves as a preparatory or sensitizing cue for the subsequent alarm blow. The second situation is during the labored breathing of a recumbent horse, in which case the sound lasts 1.0–1.8 seconds.
*Vacuum chewing* (VC)	Operational definition	Chewing with nothing in the mouth^34^.
	Functions suggested	Vacuum chewing indicates frustration in horses^32,37^. It is considered as a displacement behavior in stressful situation in other species^35,36^. This behavior has been also associated to emotions, which have a positive valence, regardless of the arousal level of individuals^38^.
*Head and body shaking* (HBSH)	Operational definition	Rapid rhythmic rotation of the head, neck and upper body along the long axis while standing with feet planted.
	Functions suggested	Stress-related head shaking is characterized by repeated rhythmic flipping motions of the head^39^.
*Avoidance/Flee attempt*	Operational definition	The head is usually held low and ears turned back. The retreat can be at any gait but typically occurs at the trot^67^.
	Functions suggested	The horse moves away from a general stressor^40^.

Regarding physiological parameters, in addition to the mean value of Heart Rate (beats/min), the heart rate variability (HRV) in time domain was collected^40^. Some of those variables referring to HRV are specifically linked to the transition toward sympathetic control of cardiac activity (standard deviation of the beat-to-beat intervals, SDRR and the square root of the mean squared differences of successive beat-to-beat intervals, RMSSD^46^). According to a general rule in HRV collection, we obtained a unique value for each of the HRV parameters (such as SDRR and RMSSD) over 5 minutes^11^.

To verify the presence of any possible variation in physiological parameters, a comparison of physiological variables between Pre-Test and Stress-Test has been made. Since these data were collected in a time domain of 5 minutes, a measure of the variability of changing features was needed. In this regard, specific formula has been elaborated in order to obtain a unique value for each physiological variable comparing their trend during the control and experimental conditions (Pre-Test *versus* Stress-Test).

$${\Delta }_{{\rm{Physiological}}{\rm{Parameter}}}={\rm{Value}}\,{\rm{of}}\,{\rm{the}}\,{\rm{parameter}}\,{\rm{during}}\,{{\rm{the}}}_{{\rm{Stress}}{\rm{Test}}}-{\rm{Value}}\,{\rm{of}}\,{\rm{the}}\,{\rm{parameter}}\,{\rm{during}}\,{{\rm{the}}}_{{\rm{PreTest}}}$$

Same formula was applied for the frequency of those behaviors which were found to vary between the two tests.

$$\begin{array}{ccc}{\Delta }_{{\rm{Behavior}}} & = & {\rm{N}}^\circ \,{\rm{of}}\,{\rm{time}}\,{\rm{the}}\,{\rm{behavior}}\,{\rm{was}}\,{\rm{displayed}}\,{\rm{during}}\,{{\rm{the}}}_{{\rm{StressTest}}}\\  &  & -{\rm{N}}^\circ \,{\rm{of}}\,{\rm{time}}\,{\rm{the}}\,{\rm{behavior}}\,{\rm{was}}\,{\rm{displayed}}\,{\rm{during}}\,{{\rm{the}}}_{{\rm{PreTest}}}\end{array}$$

All data generated or analyzed during this study are included (see Supplementary Information) while original videos are available from the corresponding author on reasonable request.

Non-parametric statistics was applied to those data that did not follow a normal distribution (KS; p < 0.05). The frequency of behaviors in Pre-Test and Stress-Test was compared via the Exact Wilcoxon’s Signed Rank Test. The Friedman Test was used to investigate the variation of behaviors, in terms of frequency, across the different minutes during the Stress-Test. The Dunnett post-hoc Test was applied to detect in which minutes the frequency of behaviors significantly differed. To check for a potential correlation between behavioral and physiological data the Spearman Test was used. Finally, via the Paired Samples T Test we compared physiological data recorded during the Pre-Test and the Stress-Test.

### Ethical statement

This study was carried out in accordance with the EU Directive 2010/63/EU for animal experiments (adopted by the Italian Animal Care Act, decree Law 26/2014). The Ethical Committee on Animal Experimentation of the University of Pisa approved the experimental design (Prot. N. 0033937/2018). Consent to participation in the test was signed by each horse owner.

## Results

Regarding the avoidance/attempt to flee pattern, 28 out of 33 horses showed at least one avoidance or attempt to flee immediately after the administration of the stimulus (inflated balloon).

### Differences in frequency of behaviors between Pre-Test and Stress-Test

To investigate the variability of behaviors, in terms of frequency of appearance for each one of them, we collected the onset of all behaviors, one per time, for all animals comparing the Pre-Test and Stress-Test. The distribution of *snore* behavior significantly differed between Pre-Test and Stress-Test (Snore_Pre-Test_ mean ± SD 0.63 ± 1.08; Snore_Stress-Test_ mean ± SD 4.18 ± 5.91; Exact Wilcoxon’s Signed Rank Test, T_SN_ = 42.50, ties = 5, n = 33, p = 0.0001) with a higher frequency of *snore* in the Stress-Test when compared to the Pre-Test. No differences were found between Pre-Test and Stress-Test regarding the frequency of *snorts* (Snort_Pre-Test_ mean ± SD 0.27 ± 0.51; Snort_Stress-Test_ mean ± SD 0.21 ± 0.59; Exact Wilcoxon’s Signed Rank Test, T_SNT_ = 37.00, ties = 20, n = 33, p = 0.523). The performance of *vacuum chewing* significantly differed between Pre-Test and Stress-Test (Vacuum chewing_Pre-Test_ mean ± SD 0.36 ± 0.89; Vacuum chewing_Stress-Test_ mean ± SD 0.90 ± 1.10; Exact Wilcoxon’s Signed Rank Test, T_VC_ = 27.00, ties = 16, n = 33, p = 0.017), with a higher frequency of the behavior during the Stress-Test. No differences were found between Pre-Test and Stress-Test regarding the frequency of *head/body shaking* (Head/body shaking_Pre-Test_ mean ± SD 0.66 ± 1.65; Head/body shaking_Stress-Test_ mean ± SD 0.81 ± 2.33; Exact Wilcoxon’s Signed Rank Test, T_HBSH_ = 32.00, ties = 22, n = 33, p = 0.928).

### Trends of variation in *Snore* and *Vacuum Chewing*

**Figure 1:**
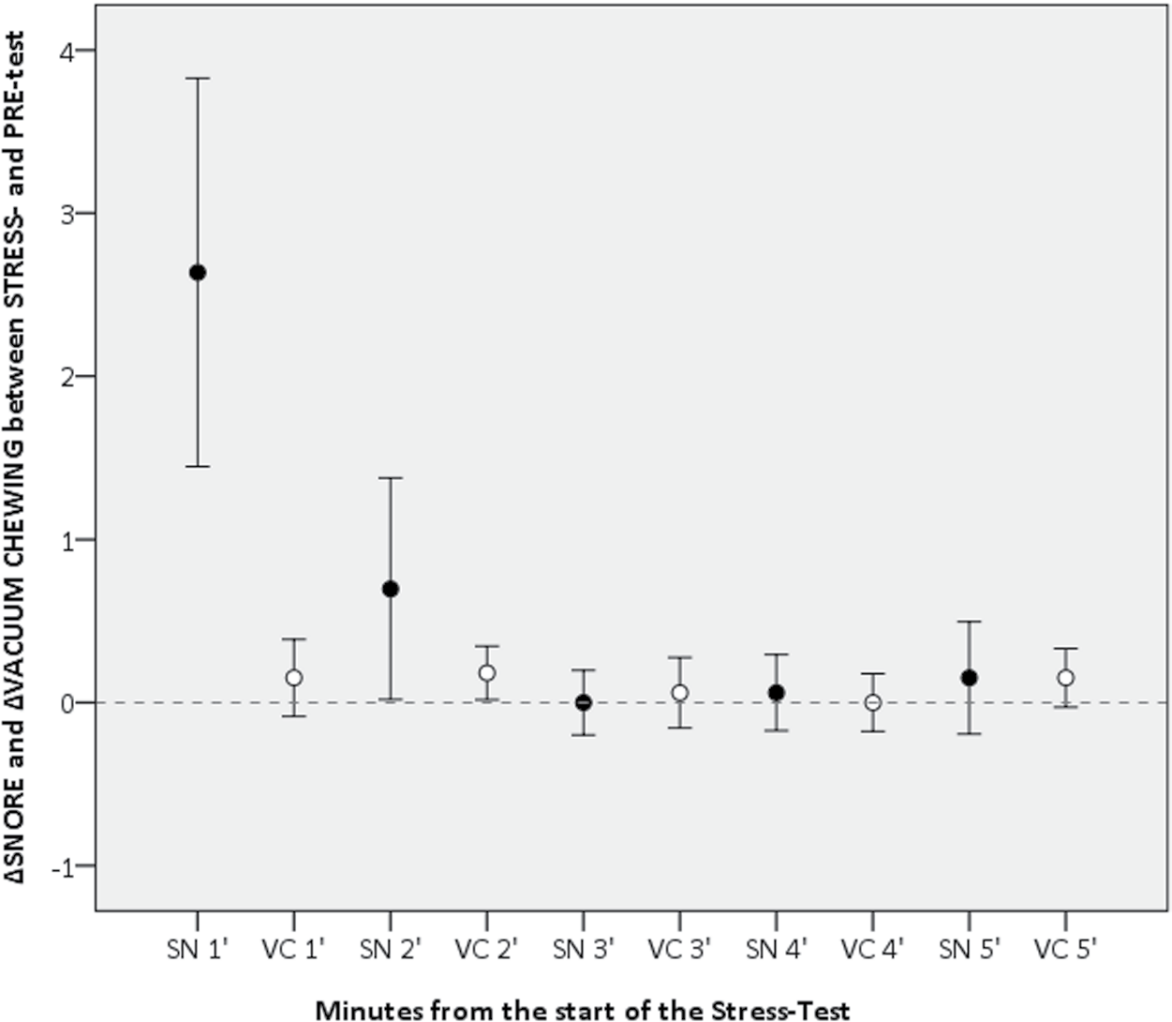
Variation in frequency of *snore* (SN, black dot) and *vacuum chewing* (VC, white dot) during each minute of the Stress-Test. Variations were calculated via the ∆ formula accounting for the rate of both behaviors comparing the Pre-Test and the Stress-Test. A clear difference between the performances of the behaviors is observable, with a peak of *snore* occurring in the first couple of minutes of the experiment, and the *vacuum chewing* constantly performed during the test.

In order to have an integrating perspective of the tendency shown by *snore* and *vacuum chewing* across the stress condition, we developed a graphical model of their performance in which the variation of frequency of both *snore* and *vacuum chewing* is shown, minute by minute during the Stress-Test (Fig. 1). The ∆ formula employed here accounts for the rate of both behaviors during the 5 minutes Stress-Test, revealing a slight difference between their patterns: the *snore* occurrence shows in fact a distinct peak in the first couple of minutes of the experiment and then it gradually wanes; on the other hand, the *vacuum chewing* frequency seems to permeate the entire test with its mild incidence, constantly affecting the whole duration of the trial. Even though these behaviors were the only ones whose frequency differed between the Pre-Test and the Stress-Test, when it came to look at the dissimilarity between each minute of the Stress-Test, a significant variation may be seen within the frequency of *snore* behavior (Friedman Test, χ^2^ = 41.5, df = 4, n = 33, p = 0.0001), while no significant differences have been found in the variation of *vacuum chewing* (Friedman Test, χ^2^ = 5.4, df = 4, n = 33, p = 0.245).

Moreover, the Dunnett post-hoc Test confirmed the strong discrepancy between the ∆*snore* in the 1′ and the 2′ minute of Stress-Test (p = 0.01), likewise the 2′ and the 3′ minute (p = 0.05). No differences were found between the 3′ and the 4′ minute (p = 1.00), or the 4′ and the 5′ ones (p = 1.00).

### Behavioral data and physiological variables

Before verifying whether the difference between the Pre-Test and the Stress-Test in terms of frequency of behaviors would have had a correspondence in physiological cardiac activity, a direct comparison between the physiological parameters values across the control and experimental conditions needed to be conducted in order to exclude from the comparison with behavioral data, those variables which remained basically unchanged during the Stress-Test.

The trend of all physiological parameters collected was compared between Pre-Test and Stress-Test. The Heart Rate (HR) and the standard deviation of the beat-to-beat intervals (SDRR) values are the only parameters which show significant difference between the Pre-Test (HR mean ± SD 45.70 ± 13.27; SDRR mean ± SD 121.25 ± 53.47; Paired Samples T Test, T = −2.613, n = 33, p_HR_ = 0.014) and the Stress-Test (HR mean ± SD 49.09 ± 11.11; SDRR mean ± SD 196.70 ± 100.95; Paired Samples T Test, T = −4.470, n = 33, p_SDRR_ = 0.0001), with a higher rate during the Stress-Test in both of them.

**Figure 2:**
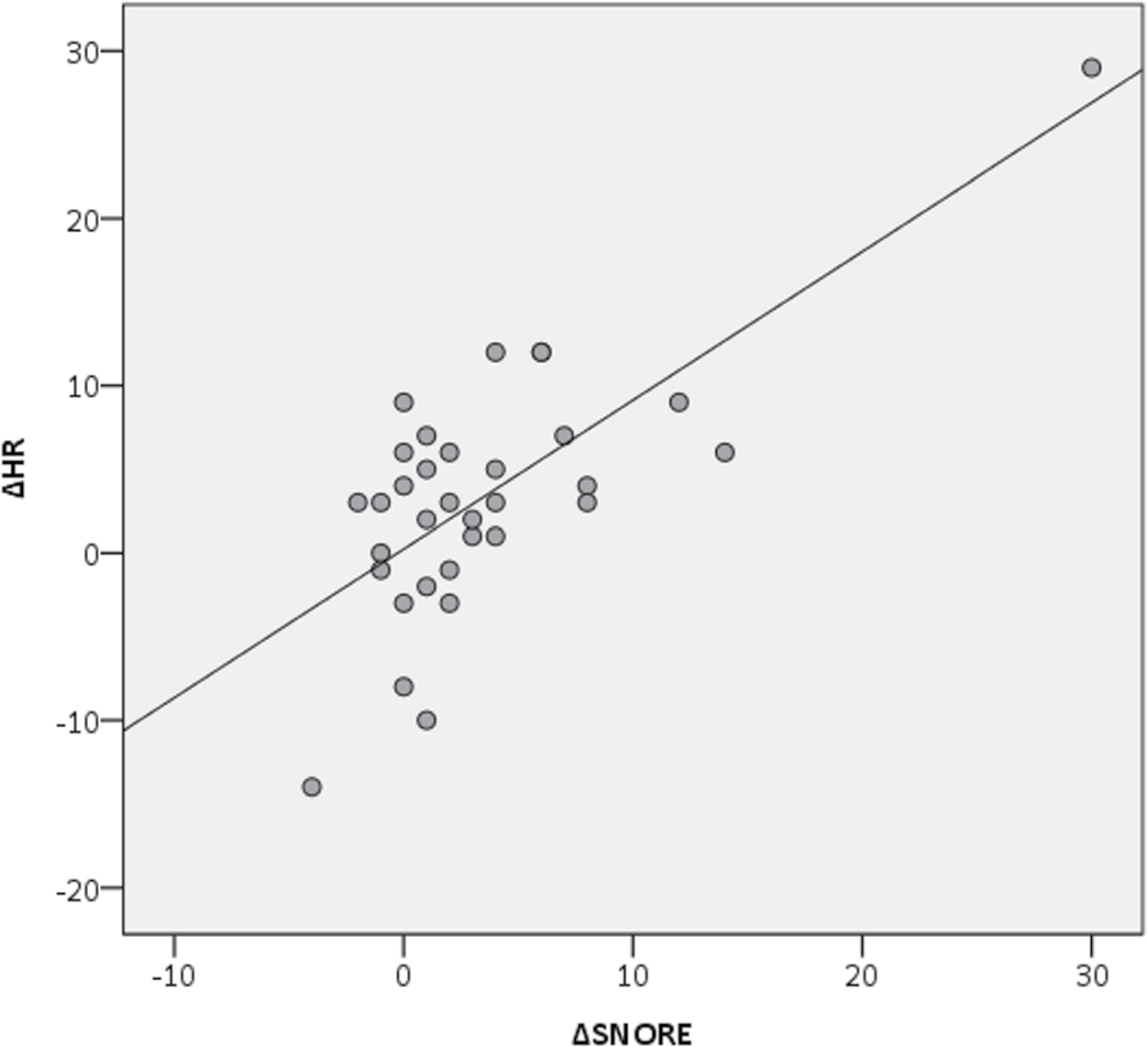
Correlation between the variation of the *snore* behavior (∆SNORE) and the variation of the heart rate (∆HR) between Pre-Test and Stress-Test (Spearman Correlation, r = 0.545, n = 33, p_∆SN∆HR_ = 0.001).

**Figure 3:**
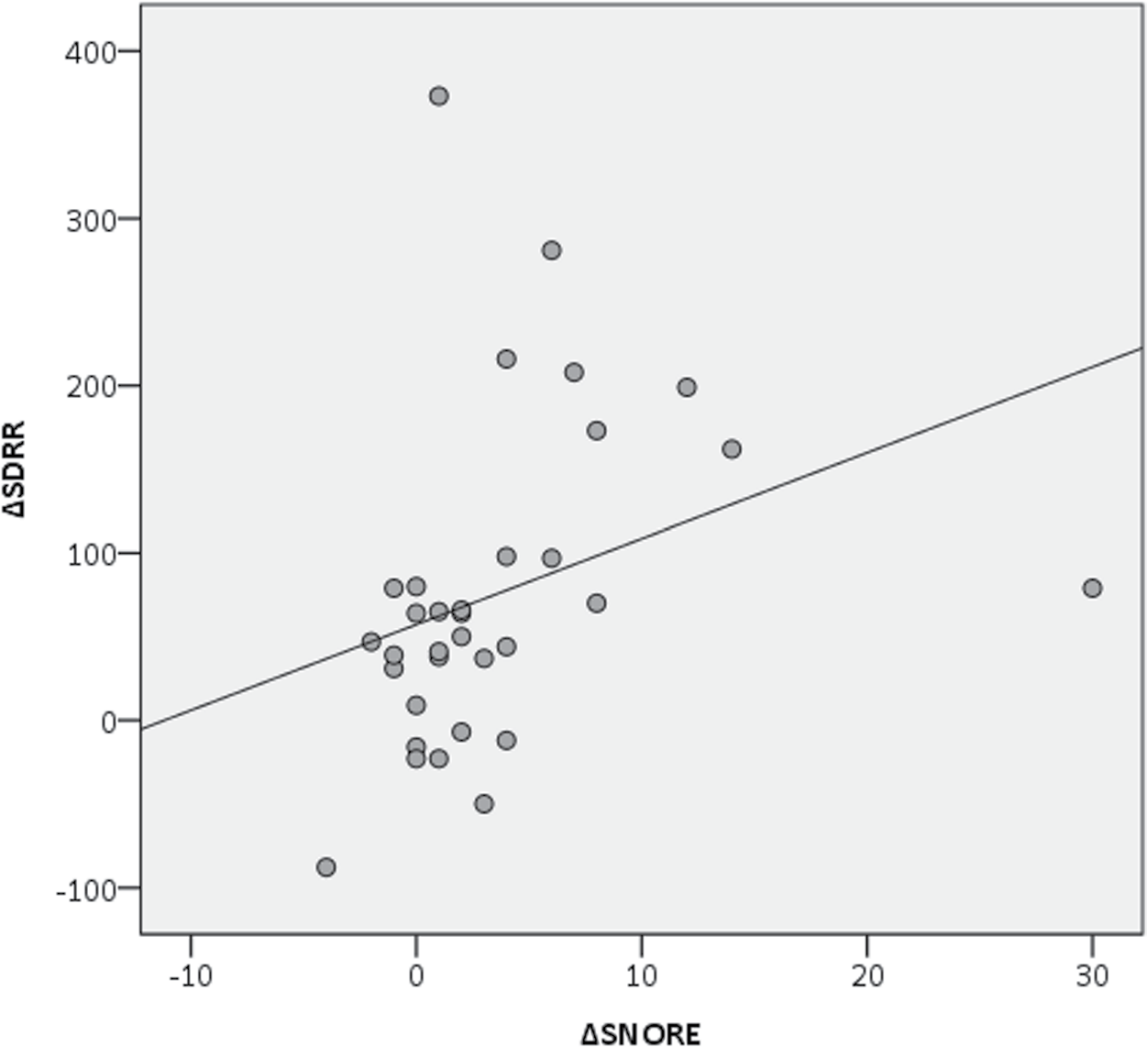
Correlation between the variation of the *snore* behavior (∆SNORE) and the variation of the Standard Deviation of R-R peak intervals, SDRR (∆SDRR) between Pre-Test and Stress-Test (Spearman Correlation, r = 0.524, n = 33, p_∆SN∆SDRR_ = 0.002).

Once established which physiological parameters increased in the stressful experimental setting, the correspondence between behavior and physiology needed to be verified. The correlation between increasing physiological variables (HR and SDRR) and increasing behavioral frequency (*snore* and *vacuum chewing*) has been conducted using a specific ∆ formula (see 2.1) which contemplates changing values, thus measuring the variation. The test revealed a positive correlation between the ∆*snore* and the ∆HR (Spearman Correlation, r = 0.545, n = 33, p_∆SN∆HR_ = 0.001; Fig. 2). In order to avoid a deceiving result due to the presence of an outlier, the test has been replicated by removing the outlier from the sample. The correlation remained statistically positive (Spearman Correlation, r = 0.500, n = 32, p_∆SN∆HR_ = 0.004). A positive correlation has been also found between the ∆*snore* and the ∆SDRR (Spearman Correlation, r = 0.524, n = 33, p_∆SN∆SDRR_ = 0.002; Fig. 3). Regarding ∆*vacuum chewing*, no correlation has been found with the ∆HR (Spearman Correlation, r = 0.093, n = 33, p_∆VC∆HR_ = 0.606), nor with ∆SDRR (Spearman Correlation, r = 0.294, n = 33, p_∆VC∆SDRR_ = 0.096).

In order to be sure that those behavioral patterns that did not differ in frequency would have not been considered as indicator of frustration in horses, we also verify the potential correlation between ∆*snorts* and ∆*head/body shaking* with physiological variation. Neither the *snorts* nor the *head/body shaking* correlated with any physiological parameters (Spearman Correlation, r = 0.133, n = 33, p_∆SNTS∆HR_ = 0.462; r = −0.177, n = 33, p_∆SNT∆SDRR_ = 0.325; r = 0.046, n = 33, p_∆HBSH∆HR_ = 0.800; r = 0.144, n = 33, p_∆ HBSH∆SDRR_ = 0.424). To exclude the possibility of a spurious result, a test was conducted to confirm the lack of correlation between the two physiological parameters (Pearson Correlation, r = 0.218, n = 33, p_∆SDRR∆HR_ = 0.223).

## Discussion

Our results show that the only behavioral pattern satisfying the two criteria postulated to consider a behavior as a reliable stress indicator was the *snore*. Snore frequency significantly increased in the Stress-Test (criterion 1) and its variation (*snore*_experimental_ minus *snore*_control_) correlated with the shifting of the physiological variables linked to heart activity (criterion 2). *Vacuum chewing* satisfied only the first criterion thus increasing after the administration of the stimulus, but it did not show correlation with any of the physiological parameters considered. *Snort* and *head/body shaking* did not satisfy either the first or the second criterion.

The function of *snore* in horses has been associated to fear for novel stimuli, probably used prior to non-vocal alarm sounds^47^. A scientific debate embraces the potential function of *vacuum chewing*, whose occurrence generally reveals a state of frustration in horses^32,37^ and it is considered as a displacement activity in some other species^35,36^.

Our results also show that the only physiological parameters that differed between the control and experimental conditions were the HR and the SDRR. The values of HR and the SDRR were higher during the Stress-Test compared to the Pre-Test. SDRR is a measure of the variability across the different R-R intervals, thus estimating the overall HRV and therefore including the contribution of both branches of the autonomic nervous system. Generally speaking, a reduction in SDRR indicates a transition toward sympathetic control over cardiac activity and, as a consequence, an increase of the stress level. It is worth noting that, contrary to what we have found, an overall decrease of SDRR should have been expected in a stressful situation.

Looking at the behavioral variation throughout the Stress-Test, a peak in ∆*snore* during the first couple of minutes appears conspicuous, probably due to the sudden appearance of the unfamiliar object, working as a sort of preparation to investigation. This immediate response is also confirmed by the heart rate (HR) in the Stress Test, which is indeed higher if compared with the Pre-Test. Unlike snore*, Vacuum chewing*, although the low frequency of performance (only 19 of 33 animals performed this behavior), is constantly enacted throughout the trial, thus minimizing the overall variation of the behavior itself during the 5-min time window. Taken together all these data suggest that *snore* and *vacuum chewing* are stress-releasing behaviors that, at the same time, indicate a stressful condition.

As in the case of self-grooming, scratching and yawning in human^36^ and non-human primates^48^, *snore* and *vacuum chewing* can be considered displacement activities. Such activities occur under circumstances in which they are apparently irrelevant to ongoing events and that seem to reflect the motivational ambivalence/frustration coming from conflict situations^35,49–51^. There is a linkage between self-directed behaviors (displacement activities) and stress levels. For example, in monkeys Duboscq *et al*.^52^ demonstrated a strong connection between self-directed behaviors and stress-induced hormones. Moreover, in the minutes following aggression monkeys (*Macaca* spp.) experience an increase of HR and self-directed behaviors (scratching, in this case)^53–55^. Yawning in adult boobies (*Sula granti*) has been explained by the ‘arousal reduction hypothesis’, which claims that this species yawns to down-regulate arousal, after external stressors disrupted its balance^56^. In a similar way, the ‘state changing hypothesis’ predicts that yawning in lemurs (*Lemur catta*) is a potential physiological enhancer associated to the transition from one behavior to another^57^. Both these hypotheses, which explicitly focus on the internal state of the animal, seem to fit with our results on self-directed behaviors in horses.

Snores, often followed by blows (which corresponds to a short very intense non-pulsed exhalation through the nostrils and is generally associated with vigilance/alarm postures^33,47^, indicate low alert context prior to the investigation of novel objects or obstacles, following the terminology proposed by Stomp *et al*.^33^. Snore, along with blowing, has been considered indicator of emotionality and fear in several studies^58,59^. Briefer and colleagues^38^ suggested that the increased time spent in vacuum chewing during controlled positive situations, as compared to negative ones, could indicate positive emotions in horses triggered by the sight of their group mate(s) coming back to the stable, following the high-arousal negative emotion triggered by group mate(s) leaving. These interpretations of *snoring* and *vacuum chewing* suggest a sort of transitional role of these behaviors, which appear to occur at the same time as the individual’s emotional state varies in order to adapt to a new condition.

The maintenance of “homeostasis” explains how the deviation from a specific set-point of a series of physiological variables may be counteracted by physiological responses whose only purpose is to restore the basal level^60^. This ability, also called resilience, is strongly adaptive^61^. In this perspective, it is not only important how rapid and efficient the recovery could be (i.e. the modulation of resilience)^62^, but also which are the behavioral strategies contributing to such recovery. If we consider *snore* as a stress-releasing behavior mainly expressed in the first two minutes of the Stress-Test to restore a basal condition, the variation of SDRR values obtained across the 5-min window is not surprising. The homeostasis obtained via the enactment of such behaviors could be physiologically expressed in a proper sympato-vagal balance. Hence, in this case, resilience skills could correspond to a prevalence of parasympathetic control that can come into play in the last minutes of the Stress-Test thus increasing SDRR values. Furthermore, the horse is a vagotonic animal, meaning that its heart rate is under vagal inhibition up to 120–140 beats/min^63,64^ and, even though the HR increased during Stress-Test in our study, it never reached such peak frequency.

## Conclusion

In conclusion, not all the self-directed behaviors considered can function as stress-releasing behaviors in horses, but only those which increased in frequency during the experimental test and whose variation correlates with the variation of specific physiological parameters. In particular, the variation of the *snore* was found to be modulated over time, contrary to the *vacuum chewing*, whose variation appears constant across the time-window considered. Then, we cannot exclude that some other behaviors could occur later, thus playing a role in stress-releasing at a delayed level.

In this study, we hypothesized that in horses the resilience ability is an adaptive strategy useful for managing everyday environmental and social challenges. The capacity to recognize specific frustration-related behaviors in horses is crucial for riders, owners and caretakers to properly read and interpret the internal state of animals and, in turns, improve their welfare.

To effectively get all the benefits coming from the combination of behavioral and physiological signals, a promising goal would be the accomplishment of more accurate HRV detection in animals, as it has been already done for humans. Indeed, a new approach of data analysis and interpretation has recently allowed to record human HRV with an interval of 30 seconds^46,65^. Applying the same method to non-human animals, it would be possible to accurately link physiological parameters and behaviors in a shorter time domain.

## Electronic supplementary material

Supplementary Table S1

Dataset1
