# Supplementary material for: Physiological outcomes of calming behaviors support the resilience hypothesis in horses
Source: Sci Rep. 2018 Nov 30;8:17501. doi: 10.1038/s41598-018-35561-7 (PMC6269543; doi:10.1038/s41598-018-35561-7)
Supplement: Supplementary file 1 — Supplementary Table S1 [file 41598_2018_35561_MOESM1_ESM.pdf]

Physiological outcomes of calming behaviors support the resilience hypothesis in horses

Chiara Scopa[1], Elisabetta Palagi[2], Claudio Sighieri[3], Paolo Baragli[3*]

1 Italian National Reference Centre for Animal Assisted Interventions, Istituto Zooprofilattico Sperimentale delle Venezie, Legnaro (Padua), Italy
2 Natural History Museum University of Pisa Calci Italy
3 Department of Veterinary Sciences, University of Pisa, Italy

Supplementary Table S1

Horse	Age	Sex
banwieu	16	G
beauty	24	F
calipso	22	G
coral	12	G
degas	14	G
dianca	22	F
doris	18	G
espana	9	F
fomelia	22	F
gitan	12	G
jim	24	G
kalief	11	G
magic	15	G
mauritz	14	G
mazad	14	G
mnoris	8	G
mr blue	18	G
mustafà	12	G
naftan	11	G
nepal	10	G
noelle	6	F
pampero	15	G
parsifal	8	G
piorum	16	G
pippo	6	G
remulus	11	G
rocky	24	G
roi	15	G
ruchel	11	G
stallone	10	G
sultan	24	G
vadoc	18	G
zefir	10	G

List of the animals included in the analyses (F = female; G = gelding).
